# Caregivers’ perception of ageing, perceived health and strain in caregiving – The preliminary cross culture analysis

**DOI:** 10.1002/alz.084925

**Published:** 2025-01-09

**Authors:** Frank HY Lai, Andrew Man‐hong Siu, Eddie Yip‐kuen Hai, Stephanie Whittington, Cynthia Yuen‐yi Lai, Wallace Chi‐ho Chan, Ben Chi‐bun Yip

**Affiliations:** ^1^ Northumbria University, Newcastle United Kingdom; ^2^ The Mental Health Research Centre, The Hong Kong Polytechnic University, Kowloon Hong Kong; ^3^ Department of Health Sciences, College of Health, Medicine and Life Sciences, The Brunel University, London United Kingdom; ^4^ Faculty of Health Sciences and Wellbeing, The Sunderland University, Sunderland United Kingdom; ^5^ Department of Social Work, Education and Community Wellbeing, Faculty of Health and Life Sciences, The Northumbria University, Newcastle United Kingdom; ^6^ Department of Rehabilitation Sciences, The Hong Kong Polytechnic University, Kowloon Hong Kong; ^7^ School of Medical and Health Sciences, Tung Wah College, Kowloon Hong Kong

## Abstract

**Background:**

Understanding how caregivers perceive their own aging process is essential for addressing their well‐being and the quality of care they provide. This study aimed at exploring the relationship between caregivers’ self‐perception of ageing in relation to their strain experienced in caregiving, and their perceived physical and mental health symptoms. Moreover, to examine if the strain experience and perceived health condition were key predictors to their self‐perception of ageing. Additionally, to analyze how demographic variables, such as gender and cultural background, would impose influence on self‐perception of ageing

**Method:**

A questionnaire was developed in collect data on the variables, including self‐perceptions of ageing (SPA), caregivers' perceived strain (PS), caregivers physical health symptoms (PHS), and mental health symptoms (MHS).

**Result:**

60 spousal caregivers, 33 male and 27 female with (24 Western, 24 Asian, and 12 Black Asian) participated in a cross‐sectional survey. There was a strong negative correlation between the self‐perception of ageing and caregivers’ strain (r = −.55, *p* < .001), physical symptoms (r = −.67, *p* < .001), and mental health symptoms (r = −.36, *p* < .001). Results of multiple regression analysis showed that caregivers’ strain, physical symptoms, and mental health are good predictors of self‐perception of ageing, and the regression equation account for a significant proportion of variance in self‐perception of ageing (R^2^ = .56, Adjusted R^2^ = .53).

**Conclusion:**

Cultural background imposes influence on self‐perception of ageing, and there are significant interactions between gender and cultural background. Post‐hoc multiple comparisons using Bonferroni tests showed that Western had significantly higher self‐perception of ageing scores than the other two culture groups, while the differences between Asian or Black Asian group are significant.

